# Prediction model study of overweight and obesity in preschool children with allergic diseases from an ecological perspective

**DOI:** 10.1186/s12887-021-02515-4

**Published:** 2021-01-25

**Authors:** Jeong-Won Han, Da-Jung Kim

**Affiliations:** College of Nursing Science, Kyung Hee University, 26, Kyunghee-daero, Dongdaemun-gu, Seoul, 02453 South Korea

**Keywords:** Allergy, Children, Overweight, Obesity

## Abstract

**Background:**

Allergic diseases have a high incidence in childhood and a high chance to be carried over into adulthood unless appropriately treated during childhood, it is important that healthcare providers actively manage these diseases. This study was to identify multidimensional factors that affect weight gain in preschool children with allergic diseases.

**Methods:**

The overweight and obesity prediction model for children with allergic diseases was analyzed using multiple logistic regression analysis and a decision tree model and the present study was a secondary data analysis study that used data from the Panel Study on Korean Children conducted by the Korea Institute of Child Care and Education.

**Results:**

The significance of this study is identify multidimensional factors that affect weight gain in preschool children with allergic diseases, which found that children (gender, sitting time during weekdays, sleeping hours during weekends,), parent (education level, mother’s job, quality of the home environment), local community (convenience of local community facilities, satisfaction level with local community facilities, quality of childcare in the local community) characteristics affected overweight and obesity at multidimensional levels as risk factors.

**Conclusions:**

The significance of this study is identify multidimensional factors that affect weight gain in preschool children with allergic diseases using the data of the Panel Study on Korean Children, which found that children, parent, local community characteristics affected overweight and obesity at multidimensional levels as risk factors.

**Supplementary Information:**

The online version contains supplementary material available at 10.1186/s12887-021-02515-4.

## Background

Allergic diseases are caused by the hypersensitive response of the immune system to foreign antigens, and representative allergic diseases in children include atopic dermatitis, asthma, and allergic rhinitis [1]. Since 1990, the prevalence of atopic dermatitis in children has continuously increased in many regions including East Asia, Western Europe, Northern Europe, and Africa [2]. The global prevalence of asthma and allergic rhinitis is also above 35.1% [3]. In Korea, 10-year trends in asthma remained low and stable, meanwhile, 10-year trends in allergic rhinitis significantly increased or remained stable [4].

Recent reports have indicated that allergic diseases are associated with weight gain, thus emphasizing the importance of weight management as well as symptom management in children with allergic diseases [5]. According to the relevant studies [6], allergic diseases and weight gain may be induced by similar foods, hence their correlation. It is also suggested that the accumulation of mast cells may cause hormonal changes and chronic airway inflammation, leading to allergic diseases. Moreover, it is suggested that children with allergic diseases may have lower levels of physical activity due to the diseases, thus increasing the risk of obesity. A study with Taiwanese children [7] also reported that an increase in body mass index and the prevalence of allergic diseases were related depending on the gender of children, and a study using the data of the National Health and Nutrition Examination Survey in the United States [8] also showed a correlation between allergic diseases and obesity depending on children’s gender and physical activity level.

Children are significantly influenced by their parents and local community environments, as well as individual characteristics, it is necessary to identify various factors in relation to children’s weight gain from an ecological perspective [9]. First, there were differences in weight gain based on children’s gender when weight gain factors were investigated at the individual level [10, 11]. Reduced physical activity and longer sitting time are factors related to weight gain in children [12–15]. Particularly, “screen time” refers to the duration of time spent in front of electronic monitors [16]. An increase in screen time induces media addiction, leading to children’s weight [17]. In addition, children’s daily intake of vegetables or fruits had a positive effect on symptom control of allergic diseases [18], and was found to affect the prevention of obesity [19]. In a meta-analysis by Fatima et al. [20], children with shorter sleeping hours had a risk of overweight or obesity 2.15 times higher than those with sufficient sleeping hours. It is understood that children with insufficient sleep have lower physical activity levels during the daytime due to fatigue [21], and a decline in willingness to exercise, leading to weight gain [22].

Regarding children’s weight gain, the home environment factor should be considered as well. Families with low-income levels have relatively limited options for healthy foods or activities for weight management [23–25]. According to a study with Swedish children and parents [26], children’s weight gain was correlated with the education level of their parents. Parents with lower education levels had a low ability to manage the health of their children and lower access to resources. Furthermore, because working mothers are limited in caring for their children, their children tended to consume more snacks, particularly high-calorie foods such as fast food, which affects children’s weight gain [27]. Particularly, parents with high depression levels tend to have heavier children. This is because severely depressed parents are not able to sufficiently control their own behavior, leading to behavioral problems such as binge eating [28]. This naturally allows their children to acquire unhealthy eating habits; hence, the depression level of parents is an influencing factor on children’s weight gain [29].

Children’s weight gain is also related to local community environments. A local community with a high-quality environment for the growth of children fosters a setting in which parents and children engage in various outdoor activities, and such long activity periods reduce children’s sitting time [30, 31]. A high poverty rate in the local community limits the provision of parks and sports facilities for children’s physical activities [32]. Accessibility to local community facilities such as playgrounds and parks is related to an increase in children’s physical activities [33–35]. The number of internet cafes in the local community can be a factor that increases the potential for children to gain weight, because they end up sitting in front of computer screens [36]. Mellor et al. reported [37] that elementary school students living close to fast-food restaurants had a high prevalence of obesity, which was because children had easy access to fast-food restaurants and thus had more opportunities to consume high-calorie foods, leading to weight gain.

An increase in obesity incidence among children with allergic diseases could cause various problems, such as increases in medical costs, family burdens, and social costs; a decrease in children’s quality of life; and a higher incidence of adult diseases. However, preceding studies tended to focus on the individual factors related to children’s weight gain, although it is related to a combination of family and community factors. Thus, the present study sought to identify factors that can be used for the prediction of overweight and obesity in children with allergic diseases from an ecological perspective, with the aim of providing healthcare providers who treat allergic diseases with fundamental data to guide interventions for weight management in children.

## Methods

### Study design

The present study was a secondary data analysis study that used data from the Panel Study on Korean Children conducted by the Korea Institute of Child Care and Education.

### Participants and sampling

The subjects of this study were parents and children who participated in the 10th Panel Study on Korean Children [38]. The Panel Study on Korean Children focuses on families (both children and parents) as well as local community environments. In the present study, the data of 299 parents and 9-year-old children who answered “Yes” to the following health survey questions for children were analyzed: “Have you been diagnosed with allergic diseases (atopic dermatitis, asthma, or allergic rhinitis) by a doctor?” and “Have you been treated for allergic diseases (atopic dermatitis, asthma, or allergic rhinitis) during the past 12 months?” After adjustment of gender and age based on the Korean National Growth Charts proposed by the Korea Centers for Disease Control and Prevention (2017) [39], body mass index (BMI) was applied to the finally selected children for classification into groups as follows: underweight (< 5%), normal weight (5–85%), overweight (85–95%), and obese (95% or higher).

### Measurement

The questionnaire used in this study was developed for the Panel Study on Korean Children by the Korea Institute of Child Care and Education (Supplement 1).

### Data collection

The data of the present study were obtained from the homepage of the Panel Study on Korean Children (http://www.kicce.re.kr) [38]. The 10th year data of the Panel Study on Korean Children was downloaded after approval from the panel study team. The downloaded data were provided after removing sensitive information (in other words, any information prescribed by Presidential Decree, including ideology, belief, admission to or withdrawal from a trade union or political party, political opinions, health, sexual life, and other personal information that is likely to significantly threaten the privacy of any data subject, genetic information from genetic tests, or crime history data) according to Article 23 of the Personal Information Protection Act. In addition, data on local community poverty rates were downloaded from the recipient data of the National Basic Livelihood Security System (http://kosis.kr/index/index.do) by administrative district (si, gun, and gu), which was open to the public by Statistics Korea (www.kostat.go.kr). Regarding the numbers of internet cafes, fast-food restaurants, and public sports facilities, the data, by administrative district (si, gun, and gu), that Statistics Korea (www.kostat.go.kr) and the National Tax Service (https://www.nts.go.kr) disclosed to the public were utilized.

### Data analysis

Collected data were analyzed using SPSS-WIN Version 23.0. Subjects’ sociodemographic characteristics were expressed by real numbers, percentages, mean values, and standard deviations, while the overweight and obesity prediction model for children with allergic diseases was analyzed using multiple logistic regression analysis and a decision tree model. Logistic regression could build a model with dichotomous outcome and has been proven as a powerful algorithm. Logistic regression has been well studied and used in applications on overweight and obesity [40]. Decision tree is a technique and has had many successful applications to real-world problems. Because the output of the decision tree can be organized in the form of a tree or rules, it is easy to understand the results for decision trees. Moreover, a decision tree has the ability to build models using datasets including numerical and categorical data.

## Results

### General characteristics of subjects

Regarding subject characteristics, the mean age of fathers was 41.78 ± 3.47 years, and the majority of fathers were junior college graduates (138 people, 46.0%), and managers or company employees (145 people, 48.4%). On the other hand, the mean age of mothers was 39.33 ± 3.39 years, and most mothers were college graduates (135 people, 45.2%), and homemakers (150 people, 50.2%). The mean household income of subjects was 546.47 ± 75.4200 KRW. The children comprised 170 boys (57.0%) and 129 girls (43.0%). The majority of them lived in Gyeonggi-do and Incheon (97 people, 32.4%), followed by Busan/Ulsan/Gyeongsangnam-do (56 people, 18.8%), Chungcheong-do /Gangwon-do (51 people, 16.9%), Seoul (32 people, 10.7%), Gwangju/Jeolla-do (33 people, 11.0%), and Daegu/Gyeongsangbuk-do (31 people, 10.3%). Of all 299 children, 96 were overweight and obese, and 203 were of normal weight or underweight (Table 1).

**Table 1:** General characteristics of subjects

Variables	Category	n	%
Father’s age (yr)	< 40	108	36.1
(M ± SD = 41.78 ± 3.47)	40–45	120	40.0
	45<	71	23.9
Mother’s age (yr)	< 40	156	52.2
(M ± SD = 39.33 ± 3.39)	40–45	120	40.0
	45<	23	7.8
Father’s education	Under high school	75	25.0
	College	138	46.0
	Over bachelor’s degree	87	29.0
Mother’s education	Under high school	64	21.3
	College	135	45.2
	Over bachelor’s degree	100	33.5
Father’s occupation	Manager or white collar job	145	48.4
	Service sector or sales person	44	14.6
	Engineer or machine fabricators	46	15.3
	Others	65	21.7
Mother’s occupation	Manager or white collar job	123	41.0
	House wife	150	50.2
	Others	26	8.8
Family income (million won)	< 400	100	33.5
(M ± SD = 546.47 ± 475.42)	≥400	208	69.5
Sex of child	Male	170	57.0
	Female	129	43.0
Residence	Seoul	32	10.7
	Gyeonggi-Incheon	97	32.4
	Chungcheong-do/Gangwon-do province	51	16.9
	Daegu/Gyeongsangbuk-do province	31	10.3
	Busan/Ulsan/Gyeongsangnam-do province	56	18.8
	Gwangju /Jeolla-do province	33	11.0
BMI status	Over weight or obesity	96	32.1
	Normal or low weight	203	67.9

*M* Mean, *SD* Standard deviation, *BMI* Body mass index

### Descriptive analysis of variables

Of the 299 children, 96 children were overweight and obese, while 203 children were of normal weight or underweight. Descriptive analysis results for factors related to overweight and obesity in children were as follows Table 2.

**Table 2:** Descriptive analysis of variables

Variables	Over weight or obesity		Normal or low weight	
	M	SD	M	SD
Child factors				
Media addiction	25.33	5.99	24.62	6.023
Average sedentary time during weekday	4.10	1.19	3.53	1.11
Average sedentary time during weekend	5.29	1.60	4.17	1.54
Average sleeping time during weekday	9.16	0.60	9.28	0.69
Average sleeping time during weekend	9.19	0.74	9.64	0.94
Satisfaction of time use	2.26	0.34	2.34	0.39
Dietary assessment score	1.67	0.31	1.87	0.34
Family environment factors				
Father’s depression	2.00	0.81	1.85	0.73
Mother’s depression	1.94	0.75	1.71	0.68
Quality of home environment	1.15	0.08	1.16	0.08
Community environment factors				
Poverty rate	2.78	1.15	2.73	1.20
Number of PC room (internet cafe)	26.16	4.88	26.37	4.76
Number of fast food store	203.96	26.83	201.11	26.70
Number of public athletic facilities	2.60	1.71	2.69	1.54
Convenience of community	3.27	0.71	3.30	0.68
Satisfaction of community	3.99	1.03	4.04	0.99
Quality of parenting in community	3.44	0.57	3.57	0.59

*M* Mean, *SD* Standard error

### Logistic regression analysis of overweight and obesity in children

Factors related to overweight or obesity in children with allergic diseases are shown in in Table 3. The goodness of fit test for Model 3, including individual factors for children, parents and household factors, and local community factors, was found to be significant (χ^2^ = 8.11, *p =* 0.023). Probability for overweight and obesity in children was higher in boys (OR = 5.66, 95% CI = 4.48–7.05); the mean sitting time during weekdays was longer (OR = 2.81, 95% CI = 1.81–4.35); mean sitting time during the weekend was longer (OR = 2.83, 95% CI = 2.06–3.88); mean sleeping hours during weekends was shorter (OR = 0.51, 95% CI = 0.31–0.83); and dietary habits evaluation score was lower (OR = 0.20, 95% CI = 0.05–0.81). In addition, the probability for overweight and obesity in children was higher when mothers had lower education levels (OR = 0.37, 95% CI = 0.22–0.63); mothers still have jobs (OR = 5.87, 95% CI = 2.39–14.38); the quality score of the home environment was lower (OR = 0.88, 95% CI = 0.80–0.96); the convenience of local community facilities was lower (OR = 0.61, 95% CI = 0.30–0.97); satisfaction level with local community facilities was lower (OR = 0.40, 95% CI = 0.21–0.77); and the quality of childcare in the local community was poorer (OR = 0.56, 95% CI = 0.42–0.86). Regarding model 3, logistic regression analysis found that the model had 88.2% for sensitivity, 68.8% for specificity, and 81.9% for classification accuracy.

**Table 3:** The influencing factors on overweight and obesity of child with allergy disease

Variables	Model 1						Model 2						Model 3					
	B	SE	OR	*p*	95% CI		B	SE	OR	*p*	95% CI		B	SE	OR	*p*	95% CI	
					lower	upper					lower	upper					lower	upper
Sex of child	0.47	0.42	3.13	<.001	1.71	5.18	0.45	0.53	5.82	<.001	3.14	7.90	0.50	0.59	5.66	<.001	4.48	7.05
Media addiction	0.01	0.02	0.98	.657	0.93	1.04	0.01	0.03	0.98	.609	0.92	1.04	0.01	0.03	0.99	.906	0.93	1.06
Average sedentary time during weekday	0.71	0.16	2.04	<.001	1.47	2.82	0.92	0.20	2.53	<.001	1.69	3.78	0.30	0.22	2.81	<.001	1.81	4.35
Average sedentary time during weekend	0.64	0.11	1.90	<.001	1.52	2.38	0.93	0.14	2.54	<.001	1.90	3.41	0.14	0.16	2.83	<.001	2.06	3.88
Average sleeping time during weekday	−0.44	0.29	0.85	.127	0.58	2.74	−0.44	0.32	0.83	.167	0.56	2.94	−0.58	0.35	0.70	.095	0.55	0.95
Average sleeping time during weekend	−0.67	0.21	0.50	.002	0.33	0.77	−0.66	0.24	0.51	.005	0.32	0.82	−0.66	0.24	0.51	.007	0.31	0.83
Dietary assessment score	− 0.41	0.53	0.65	.438	0.23	1.89	−0.44	0.66	0.23	.029	0.06	0.86	−0.58	0.70	0.20	.024	0.05	0.81
Father’s education status							−0.32	0.21	0.72	.129	0.47	1.09	−0.27	0.22	0.75	.223	0.48	1.18
Mother’s education status							−0.89	0.24	0.41	<.001	0.25	0.66	−0.98	0.27	0.37	<.001	0.22	0.63
Mother’s employment status							0.30	0.38	3.68	.001	1.72	7.89	0.17	0.45	5.87	<.001	2.39	14.38
Family income							−0.01	0.01	0.99	.186	0.99	1.00	−0.01	0.01	0.99	.134	0.99	1.00
Father’s depression							0.36	0.23	0.69	.118	0.43	1.09	0.29	0.25	0.74	.245	0.44	1.22
Mother’s depression							0.21	0.27	0.80	.425	0.47	1.37	0.26	0.29	0.76	.366	0.43	1.36
Quality of home environment							−0.12	0.04	0.88	.003	0.81	0.96	−0.12	0.04	0.88	.006	0.80	0.96
Poverty rate													0.06	0.19	1.07	.723	0.73	1.56
Number of PC room (internet cafe)													0.07	0.04	0.99	.872	0.91	1.08
Number of fast food store													0.01	0.08	1.00	.927	0.98	1.01
Number of public athletic facilities													0.08	0.12	1.08	.493	0.85	1.39
Convenience of community													−0.20	0.50	0.61	.017	0.30	0.97
Satisfaction of community													−0.90	0.33	0.40	.006	0.21	0.77
Quality of parenting in community													−0.59	0.34	0.56	.044	0.42	0.86

*B* Standard regression, *SE* Standard error, *OR* Odds ratio, *95% CI* 95% confidential interval, Model 1 = child factors (model fit: χ^2^ (*p*) = 19.05(.015), −2Log likelihood = 261.04, Cox & Snell’s R^2^ = 0.32, Nagelkerke R^2^ = 0.44), Model 2 = model 1 + family and parents factors (model fit: χ^2^ (*p*) = 14.36(.045), −2Log likelihood = 219.47, Cox & Snell’s R^2^ = 0.41, Nagelkerke R^2^ = 0.57), Model 3 = model 2 + community factors (model fit: χ^2^ (*p*) = 8.11(.023), −2Log likelihood = 206.01, Cox & Snell’s R^2^ = 0.40, Nagelkerke R^2^ = 0.60)

### A decision tree analysis on overweight and obesity in children

**Fig. 1:**
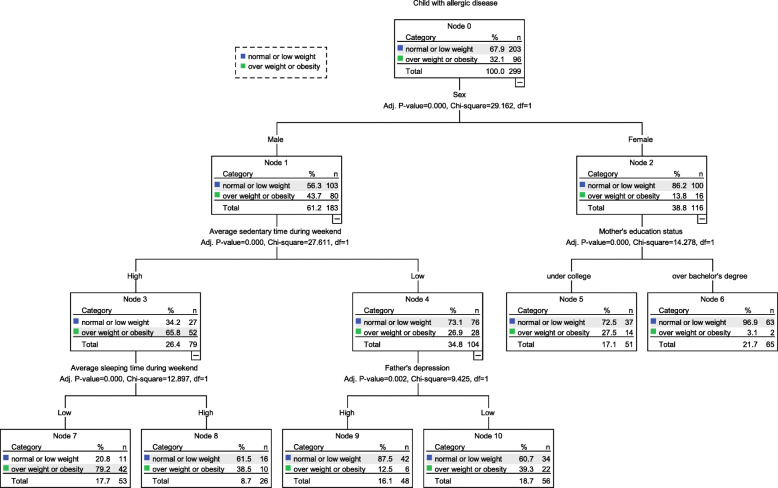
The result of decision tree

The results of the decision tree analysis are shown in in Fig. 1. Of the nodes in the structure of the decision tree, those with the highest probability for overweight and obesity in children with allergic diseases were when children were boys, when mean sitting time during the weekend was longer, and when mean sleeping hours during the weekend were shorter, which increased to 79.2% in probability after classification from 32.1% in probability before classification. The model had 94.6% for sensitivity, 43.8% for specificity, and 78.2% for classification accuracy.

## Discussion

The present study aimed to identify factors related to overweight and obesity in children with allergic diseases and to construct a prediction model; thus, these will be the focus of this discussion.

First, the logistic regression analysis model will be discussed. From an individual perspective, the present study showed that boys with allergic diseases had a relatively higher weight gain than girls, which was similar to the results of studies with Japanese children [11] and Taiwanese children [7] that indicated that boys had a higher degree of weight gain than girls. Such results suggest that girls eat less regularly and form a positive dietary attitude at a younger age than boys do, resulting in a relatively less degree of weight gain compared to boys [41, 42]. In addition, the present study found that the sitting time and sleeping hours of children correlated with weight gain, which was similar to a preceding study [17] that reported that longer screen time, most closely related to children’s sitting time such as the time spent in front of electronic monitors including TVs, computers, videos, electronic entertainment machines, portable electronic game machines, and smartphones, was correlated with children’s weight gain. Similarly, Taheri [21] focused on the relationship between shorter sleeping hours and obesity and reported that a shorter mean sleeping time was correlated with a higher incidence of overweight and obesity in children. These results indicate that longer sitting time would not only cause children to reduce physical activities, but also induce an excessive intake of foods during the screen time due to their exposure to food [43]. In addition, a reduction in sleeping hours impairs the negative feedback loop of the hypothalamic-pituitary-adrenal axis due to an increase in cortisol, interfering with appetite control, which increases food intake and results in weight gain [44]. Moreover, the present study found that the dietary habits of children with allergic diseases correlated with their weight gain, which was similar to a study in Taiwan [7] as well as one in the United States [8] using data of the National Health and Nutrition Examination Survey. Chronic airway inflammation tends to be induced in children with allergic diseases when they have an increase in intake of snacks with high salt levels and high calories, which exacerbates their allergic symptoms in addition to the accumulation of mast cells, and which, in turn, limits their physical activities due to an exacerbation of the symptoms, causing them to gain weight [6]. Especially, recent report has indicated the one of major risk factors for school accidents is impaired sleep and sleep problems increase school accidents related to allergic diseases [45]. Therefore, healthcare providers should pay attention to weight management in preschool children with allergic diseases, develop a weight management program for each gender, and plan to foster an environment enabling children to reduce sitting time while increasing the quality and hours of sleeping together with parents. Besides, healthcare providers should provide children and parents with interventions and education related to dietary habits for children to acquire healthy eating habits.

Second, when considering family and parent factors related to children’s weight gain, mothers’ lower level of education correlated with a higher incidence of overweight and obesity in children, which was similar to a study [26] with Swedish children and parents that reported a correlation between children’s weight gain and parents’ education level. These results support those of a previous study, which reported that parents with a low education level were less able to access resources on children’s health management and had less time to engage in physical activities with their children, negatively affecting children’s weight gain. Similarly, another study indicated that parents with a high education level paid more attention to children’s health, had higher access to health-related information, and had a better understanding of such information, enabling them to foster healthy living environments for their children, which resulted in a low incidence of obesity [46]. In addition, the present study found that children of currently working mothers had a relatively higher incidence of overweight and obesity than those of homemaker mothers, which was similar to a preceding study by Hawkins et al. that reported [27] a correlation between the occupation of mothers and children’s weight gain. Such results reconfirmed that, when mothers have a limitation in caring for their children due to their work, children tend to consume more snacks, particularly foods with high calories such as fast foods, influencing the incidence of overweight and obesity in children. Moreover, it was also found that higher levels of depression in parents and a lower quality home environment affect children’s overweight and obesity. Severe depression in parents degrades their own behavioral control ability, causing behavioral problems such as binge eating [28], which naturally influences their children to acquire similar unhealthy eating habits; Thus, severe depression in parents is an influencing factor on weight gain in their children [29]. Moreover, since it was caused by less interaction between parents and children, parental depression and quality of the home environment were found to be important factors. Hence, when caring for children with allergic diseases, healthcare providers should pay attention to various environments of children rather than only focusing on their characteristics. Particularly, healthcare providers should educate parents about the importance of weight management in children. Furthermore, such education should be personalized depending on the education level of the mother and on whether the mother has a job [47]. Depressed parents tend to blame children for their behaviors by perceiving them negatively without appropriate intervention, giving children a negative influence on their behaviors [5]; Thus, a health management program for children should entail a procedure to check the emotional status of their parents.

Third, considering local community factors, the present study found that children living in areas with less convenience and satisfaction with local community facilities had a higher probability of overweight and obesity. Such results showed that parents with higher accessibility and satisfaction with local community facilities more frequently use local community facilities, accordingly indicating a higher chance for children to engage in physical activities in the local community [33]. Moreover, the present study found that a lower quality of childcare in local communities was associated with a higher probability of overweight and obesity in children. This suggests that a local community with a high-quality environment for children’s growth could foster an environment enabling parents and children to engage in various outdoor activities in the local communities, and such increased activities could be helpful for weight loss through the reduction of children’s sitting time [30, 31]. To promote the health of children with allergic diseases, it is important to promote children’s physical activities in the local community through making parks and playgrounds for children’s physical activities. In addition, healthcare providers should systematically develop a program to provide information about facilities for promotion of children’s physical activities and collaborate with the local community, and also participate in policy establishments and guideline development to promote the expansion of spaces and addition of facilities in the local community and relevant educational institutions (e.g., daycare centers, kindergartens, etc.), as well as social advertising in order to promote children’s physical activities.

Lastly, regarding factors related to children’s overweight and obesity based on the decision tree analysis, the present study found that the incidence increased in boys by 43.7%, in children with a longer mean sitting time during the weekend by 65.8%, and those with a shorter mean sleeping hour during the weekend by 79.2%. In the present study, the decision tree analysis showed relatively lower results regarding sensitivity, specificity, and accuracy than the logistic regression analysis. However, the logistic regression analysis and the decision tree analysis identified commonly significant factors such as children’s gender, sitting time during the weekend, and sleeping hours during the weekend. Thus, healthcare providers who care for children with allergic diseases should evaluate such factors initially.

## Conclusions

The present study was to identify multidimensional factors that affect weight gain in preschool children with allergic diseases using the data of the Panel Study on Korean Children, which found that children, parent, local community characteristics affected overweight and obesity at multidimensional levels as risk factors. The present study should be meaningful, because it identified factors related to local communities as well as children and parents, indicated the importance of identifying multidimensional risk factors related to the incidence of overweight and obesity in children, and provided healthcare providers with fundamental data to guide interventions for weight management in children. This study provides fundamental data for intervention programs related with weight control to prevent overweight and obesity in preschool children. However, the present study is limited, in that it only utilized variables in the source data because it used the already established panel data. Thus, we suggest a more specific collection of children’s health-related data in panel surveys on children with allergies. In addition, the present study identified that children’s weight gain is inter-correlated with various factors related to children, parents, families, and local communities; thus, it is essential to focus on the development of intervention programs that employs a combination of multidimensional factors that affect weight gain in children with allergic diseases.

## Supplementary Information

**Additional file 1.**

## Data Availability

The datasets used and/or analysed during the current study are available from http://panel.kicce.re.kr/eng/index.jsp

## Abbreviations

BMI: Body mass index
MDA: Mini Dietary Assessment
MC-HOME: Middle Childhood Home
CHAID: Chi-squared automatic interaction detection
