# Supplementary material for: Prediction model study of overweight and obesity in preschool children with allergic diseases from an ecological perspective
Source: BMC Pediatr. 2021 Jan 25;21:53. doi: 10.1186/s12887-021-02515-4 (PMC7831245; doi:10.1186/s12887-021-02515-4)
Supplement: Supplementary file 1 — Additional file 1. [file 12887_2021_2515_MOESM1_ESM.docx]

Supplement 1

1. Measurement list

1) General characteristics of subjects

Of the items used in the Panel Study on Korean Children, the general subject characteristics considered in this study were age, education level, and occupation of parents, as well as household income, child’s gender, residential area, and BMI.

2) Factors related to children

(1) Media addiction

The K-SCALE for Youth Observers, an internet addiction proneness scale, was originally developed by the Internet Addiction Prevention Center ([www.iapc.or.kr](http://www.iapc.or.kr)) [1] of the National Information Society Agency, which was modified by the survey team of the Panel Study on Korean Children for preschool children. Media addiction refers to the value measured by this modified tool. The media addiction item comprised 15 questions, with each question scored on a 4-point Likert scale (1 point for “Not at all” to 4 points for “Absolutely”). A higher score meant a higher media addiction. The reliability of the tool developed was Cronbach’s α= .96, while the reliability of the present study was Cronbach’s alpha= .85.

(2) Sitting time

The sitting time item is a question developed by the survey team of the Panel Study on Korean Children, and refers to the time spent on activities involving sitting, such as doing homework, studying, reading, remote participating in online classes, watching TV, and using a smartphone or PC, compared to that spent on other daily activities. Sitting time was summed based on children’s recall of time spent on daily activities using the 24-hour Recall Method; longer hours meant longer sitting time.

(3) Sleeping hours

The sleeping hour item is a question developed by the survey team of the Panel Study on Korean Children, and refers to nighttime sleeping hours, based on children’s recall of daily schedule. Longer hours meant longer sleeping time.

(4) Assessment of dietary habits

Dietary habits were assessed using the Mini Dietary Assessment (MDA) table developed by Kim, Cho, and Lee [2], which is a scale comprising 10 questions on items such as regular meal pattern, diversity of foods consumed, intake frequency of each food group, snack intake frequency, and preference for salty foods. Each question was scored on a 5-point Likert scale (1 point for “Very poor,” 2 points for “Poor,” 3 points for “Fair,” 4 points for “Good,” and 5 points for “Excellent”), with a higher score indicating higher quality in dietary habits. The reliability of the tool in the study by Kim, Cho, and Lee [2] was Cronbach’s α= .76, while the reliability of the present study was Cronbach’s alpha= .75.

3) Factors related to the home environment

(1) Depression of parents

Parents’ depression was assessed using the Kessler depression scale (K6) [3], which comprises 6 questions scored on a 5-point Likert scale: “Never felt (1 point),” “Rarely felt (2 points),” “Sometimes felt (3 points),” “Often felt (4 points),” and “Always felt (5 points).” A higher score meant a higher degree of depression. The reliability of the developed tool was Cronbach’s α= .89, while the reliability of the present study was Cronbach’s alpha= .93 (father) or .92 (mother).

(2) Quality of the home environment

Quality of the home environment was assessed using the Middle Childhood Home (MC-HOME) observation for measurement of the environment that was developed by Caldwell and Bradley [4] for preschool children, which is composed of 8 sub-domains with a total of 59 questions as follows: 10 questions regarding Responsivity, that is, emotional and verbal responsivity to children by parents and warm parental relationship; 7 questions on the Encouragement of maturity, that is, the expectation of parents regarding their children’s mature and responsible behaviors, common rules in the family; 8 questions on Emotional climate, meaning the degree to which parents accept children’s negative expression; 8 questions on Learning materials and Opportunities that foster a learning environment, broaden the perspective of children, and show parents’ passion for learning; 8 questions regarding Enrichment, representing the intentional use of family/local community resources for the development of children; 6 questions on Family companionship, that is, participation in activities for mutual entertainment and fellowship in the family; 4 questions on Family integration, that is, whether the father (or the person who plays a “fatherly” role) can meet the needs of children; and 8 questions on the Physical environment, that is, the appropriateness of the physical environment, whether the house and its surroundings are safe, interesting, and spacious. Quality of the home environment was measured through observation by trained surveyors (19 questions) and interviews with parents (40 questions), and each question was summed with a binary scale (0 vs. 1 point). A higher score indicated a better home environment. The reliability of the developed tool was Cronbach’s α= .87, while the reliability of the present study was Cronbach’s alpha= .76.

4) Factors related to local community environments

(1) Poverty rate

The poverty rate indicates the proportion of public assistance recipients relative to the population; this was used in a study by Kramer et al. [5]. The Panel Study on Korean Children included information about the administrative districts (si, gun, and gu) of subjects’ residential areas; thus, the present study utilized data from Statistics Korea regarding the beneficiaries of the National Basic Livelihood Security System that was investigated when the 10th Panel Study was conducted. Poverty rates in the present study were calculated by dividing the number of beneficiaries of the National Basic Livelihood Security System in each administrative district by the population [6].

(2) Numbers of internet cafes, fast-food restaurants, and public sports facilities

Since the Panel Study on Korean Children included information about the administrative districts (si, gun, and gu) of subjects’ residential areas, the numbers of internet cafes, fast-food restaurants, and public sports facilities in each district (si, gun, and gu) were obtained from data collected by Statistics Korea and the National Tax Service on each administrative district [7]. Thereafter, the rate was calculated per every 100,000 people.

(3) Convenience of local community facilities

Regarding the convenience of local community facilities, the convenience of access to facilities in subjects’ local communities was evaluated, which was an item developed by the survey team of the Panel Study on Korean Children. This item was evaluated by mothers. The present evaluation tool comprised 13 questions, regarding playgrounds, parks, walking trails, theaters, exhibition and experience centers, performance centers, amusement parks, libraries, indoor sports facilities and playground, outdoor sports facilities, medical institutions, educational institutions, and public welfare facilities. Each question was scored on a 5-point scale (“Highly inconvenient: 1,” “Inconvenient: 2,” “Neutral: 3,” “Convenient: 4,” “Highly convenient: 5”). A higher score indicated a higher degree of convenience of access to local community facilities. The reliability of the present study was Cronbach’s alpha = .89.

(4) Satisfaction with local community facilities

Satisfaction with local community facilities entailed satisfaction with the use of such facilities, and the questions were created by the survey team of the Panel Study on Korean Children. This item consisted of 13 questions regarding playgrounds, parks, walking trails, theaters, exhibition and experience centers, performance centers, amusement parks, libraries, indoor sports facilities and playground, outdoor sports facilities, medical institutions, educational institutions, and public welfare facilities. Each question was scored on a 5-point scale (“Highly inconvenient: 1,” “Inconvenient: 2,” “Neutral: 3,” “Convenient: 4,” and “Highly convenient: 5”). A higher score meant a higher degree of satisfaction with local community facilities. The reliability of the present study was Cronbach’s alpha= .92.

(5) Quality of childcare in the local community

Quality of childcare in the local community, a tool developed by Suh et al. [8], was an item to evaluate whether the current residential area is appropriate for childcare; mothers evaluated the security and safety of the local community. This evaluation tool comprised 8 questions, all of which were scored on a 6-point scale (”Very poor: 1,” “Poor: 2,” “Neutral: 3,” “Good: 4,” “Excellent: 5,” and “No idea: 6”). A higher score indicated a higher quality of childcare in the local community. The reliability of the developed tool was Cronbach’s α= .78, while the reliability of the present study was Cronbach’s alpha= .71.

**2. Additional description on data analysis**

For testing the logistic regression model, its likelihood ratio was identified as a significance test of the model, and parameters (logistic regression coefficients) were estimated using the maximum-likelihood method, in which the probability to observe the estimate when a parameter estimate was given is called likelihood. The value obtained from multiplication by -2 with the logarithmic value of likelihood (-2log-likelihood) was used as an index to test how well the prediction model fitted the data. In addition, the present study used the Hosmer-Lemeshow test for examining the goodness of fit of the model, and a chi-square value was used for testing the consistency between the observed values of dependent variables and the expected values in the model. Cox and Snell’s R^2^ and Nagelkerke’s R^2^ were coefficients of determination calculated using the value of log-likelihood function, which indicated explanatory power; that is, the regression equation containing independent variables could predict dependent variables. Nodes on the decision tree analysis were split based on the CHAID (Chi-squared automatic interaction detection) algorithm, while the criterion for splitting and merging between independent variables was .05. The number of the parent nodes was 4 and 3 for the splitting criteria of the child node. The logistic regression model and decision tree model were evaluated for sensitivity, specificity, and accuracy.

**references**

1. Internet Addiction Prevention Center. Available online: <http://www.iapc.or.kr> (accessed 7 July 2020).

2. Kim WY, Cho MS, Lee HS. Development and validation of mini dietary Assessment index for Koreans.

Korean J Nutr. 2003;36(1):83-92.

3. Kessler RC, Andrews G, Colpe LJ, Hiripi E, Mroczek DK, Normand SL, Zaslavsky AM. Short screening

scales to monitor population prevalences and trends in non-specific psychological distress. Psychol Med.

2002; 32(6):959-976.

4. Caldwell BM, Bradley RH. Home inventory administration manual, 3rd ed.; University of Arkansas for

Medical Sciences, USA, 2003.

5. Kramer MR, Raskind I.G, Van Dyke ME, Matthews SA, Cook-Smith JN. Geography of adolescent obesity

in the U.S., 2007-2011. Am J Prev Med. 2016; 51(6):898-909.

6. Korean Statistical Information Service: 2017 basic livelihood security recipient. Available online:

<http://kosis.kr/index/index.do> (accessed 7 July 2020).

7. Korea Statistics Office(2017). Available online: [www.kostat.go.kr](file:///C:\Users\user\Desktop\www.kostat.go.kr) (accessed 7 July 2020).

8. Suh MH, Cho AJ, Kim YK. The Survey on the Actual Conditions of Child Care and Education in 2004: A

Survey on the Use of Child Care and Education and the Actual Conditions of Needs. Korea health society research institute. 2005.
